# Shotgun metagenomic profiling of bacterial microbiomes, metagenome-assembled genomes and antimicrobial resistance in respiratory and blood samples from Gambian children with pneumonia

**DOI:** 10.21203/rs.3.rs-8724320/v1

**Published:** 2026-04-08

**Authors:** Dam Khan, Josh L. Espinoza, Peggy-Estelle Tientcheu, Isaac Darko Otchere, Nuredin Ibrahim Mohammed, Archibald Worwui, Mark P. Nicol, Brenda Kwambana-Adams, Martin Antonio, Chris L. Dupont

**Affiliations:** Medical Research Council Unit The Gambia at the London School of Hygiene & Tropical Medicine; J. Craig Venter Institute; Medical Research Council Unit The Gambia at the London School of Hygiene & Tropical Medicine; Noguchi Memorial Institute for Medical Research; Medical Research Council Unit The Gambia at the London School of Hygiene & Tropical Medicine; Medical Research Council Unit The Gambia at the London School of Hygiene & Tropical Medicine; University of Western Australia; Liverpool School of Tropical Medicine; Medical Research Council Unit The Gambia at the London School of Hygiene & Tropical Medicine; J. Craig Venter Institute

**Keywords:** microbiome, metagenome-assembled genomes, antimicrobial resistance, Streptococcus pneumoniae, Haemophilus strains

## Abstract

Pneumonia is a leading cause of morbidity and mortality in children, with bacterial pathogens being important etiologic agents. Most microbiome studies in pneumonia use technologies with limited taxonomical resolution and few include lung aspirate or blood samples. In this study, we assessed the microbial communities of the nasopharynx, nasopharynx/oropharynx, induced sputum, lung aspirate and blood, and recovered metagenome-assembled genomes from the same sites using shotgun metagenomics sequencing of samples from children with severe and very severe pneumonia in The Gambia. Our data show that Proteobacteria and Firmicutes were the most common phyla across the body sites, and this was largely driven by *S. pneumoniae, H. influenzae/aegyptius and M. catarrhalis.* Furthermore, we observed species overlap of blood and respiratory samples with average Jaccard similarity index values ranging from 34% to 58%. We recovered 60 medium and 35 high-quality MAGs in these niches including 11 *S. pneumoniae*, 10 *H. influenzae* strains and a limosilactobacillus with less than 95% Average Nucleotide Identity to any known species in GTDB-TK. We also showed that the resistomes in our MAGs were highly species specific with more than 70% of the detected AMR genes found exclusively in a single species.

## Introduction

Lower respiratory tract infections (LRI) pose a major public health burden and remain one of the main infectious causes of death in low- and middle-income countries^1,2^. LRI, defined by the Global Burden of Disease report as pneumonia or bronchiolitis, was responsible for more than 500,000 deaths in children under five years of age worldwide in 2021^3^. A wide range of organisms cause LRI including bacteria, viruses and fungi. Among the bacterial causes of LRI in 2021, *Streptococcus pneumoniae* was responsible for the highest number of episodes and deaths with an estimated 97·9 million episodes and 505 000 deaths globally across all ages^3^. The risk factors for pneumonia include poverty, malnutrition, lack of exclusive breastfeeding and inadequate vaccination^4,5^.

Effective prevention and treatment strategies for pneumonia, including vaccination and antimicrobials, rely partly on the identification of the microbial etiologic agent of the pneumonia episode, which can be done using culture, nucleic acid amplification, or antigen testing. However, the use of these targeted detection methods limits our understanding of the complex interaction between microbes in the lungs whose interactions affect disease outcome^6^. Samples from the lower respiratory tract are difficult to obtain; therefore, the upper respiratory tract is often studied to gain insights into the microbiome and its role in disease^7^. Understanding the microbiome of the upper respiratory tract is important because it has been postulated that microaspiration is the primary route through which bacteria in the upper airways enter the lungs^8,9^, although the temporal, spatial, and regulatory steps that lead to disease are unknown. Moreover, bacterial translocation across the lung epithelium into the blood has also been reported in pneumonia cases^10^, highlighting the significance of profiling the microbiome of both the respiratory tract and blood in disease.

The relationship between the upper respiratory airway microbiome and respiratory health is gaining attention^11,12^. For instance, it has been shown that a high abundance of commensal bacterial species of the *Corynebacterium* and *Dolosigranulum* genera prime the respiratory tract in children through stimulation of innate immunity and competition for resources to restrict colonisation by pathogenic bacteria^13–15^. Conversely, an increased nasopharyngeal density of *S. pneumoniae* may be associated with respiratory illnesses in children^16,17^.

Our understanding of the microbiome has traditionally been based on findings from 16S rRNA sequencing technology^18–20^. However, this method lacks species resolution. An untargeted microbial profiling approach, such as shotgun metagenomic sequencing, will provide deeper taxonomical insight into the microbial landscape of the respiratory tract and blood during disease.

An added advantage of shotgun metagenomics over 16S rRNA sequencing is its ability to recover metagenome- assembled genomes (MAGs). MAGs are generated using machine learning methods to group assembled contigs into bins that represent individual organisms using nucleotide usage or coverage^21^. This method has become increasingly important in microbiome research for reconstructing the draft genomes of community members, including uncultured microbes^22–24^. However, many MAGs reported in published studies are of low quality and completeness ^24^. Additionally, attempts to recover MAGs from respiratory specimens remain limited. Notably, *Li et al.* constructed a comprehensive respiratory genome catalogue of 552 MAGs, although these were derived from only 20 studies^25^.

Given the common use of antibiotics regimens in pneumonia patients, profiling antimicrobial resistance genes (AMR) of MAGs in respiratory and blood samples is essential for informing targeted antimicrobial therapy. Moreover, profiling AMR genes contributes to broader efforts in combating antimicrobial resistance which is a global health concern.

In this study, we performed shotgun metagenomics and analysed nasopharyngeal swab (NPS), combined nasopharyngeal and oropharyngeal swabs (NP/OP), induced sputum (IS), lung aspirate (LA), and blood samples from children diagnosed with severe pneumonia to characterise the microbiomes across these sites. Moreover, we aimed to recover MAGs from sample metagenomes of the same specimen types and to characterise their antimicrobial resistance determinants.

## Results

### Study population demography, clinical features and specimen types

**Figure 1:**
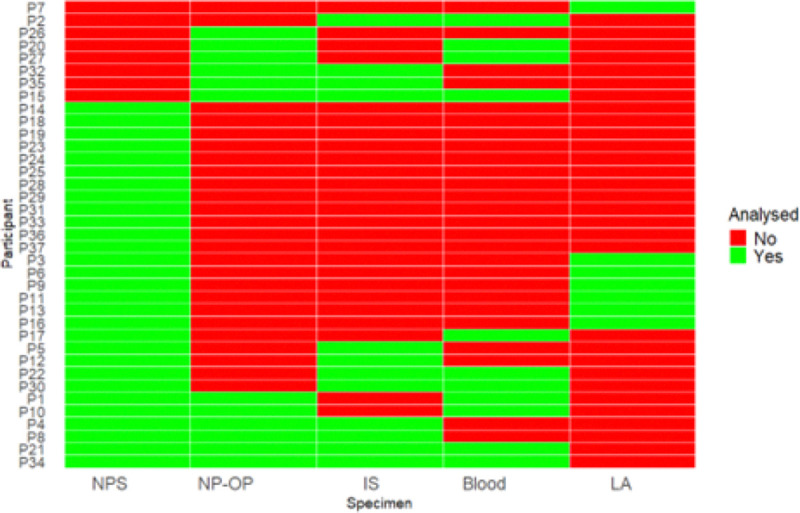
List of specimen type analysed for each of the 37 participants. Participants are sorted based on similarity in sample availability.

We analysed 71 samples from 37 children hospitalised with pneumonia and who were recruited as part of the PERCH cohort study. The demographic and clinical features of the children are presented in Table 1. The majority were male, 59% (22/37) and 41% (15/37) of the children were less than 6 months old. A total of 54% (20/37) and 51% (19/37) of the cases were fully vaccinated with the *Haemophilus influenzae* type b vaccine and the Pneumococcal Conjugate Vaccine (PCV13) respectively. Very severe pneumonia cases accounted for 57% (21/37) of the cases. NPS comprised the largest proportion of specimens accounting for 41% (29/71) (Supplementary information Table S1). Lung aspirates were the least represented sample type with seven specimens. Figure 1 shows specimens analysed from each of the 37 children. Overall, 62% (23/37) of the children provided more than one specimen type. The number of samples analysed in this study was based on availability.

### Microbiome composition of children with pneumonia

Shotgun metagenomic sequencing yielded a median of 19.4 million reads per sample. On average, 85% of the reads were human-derived. After quality trimming and removal of human reads, the median read count per sample was 1.3 million. All ecological analyses, including relative abundance bar plots, diversity measures, and differential abundance testing, were performed using the SLC relative abundance and prevalence across samples. The *Haemophilus aegyptius* (a *H. influenzae* biotype) MAG obtained in our dataset clustered at the 95% ANI with *H. influenzae* MAGS. This SLC is designated as *Haemophilus influenzae/aegyptius*. In SLCs that lack species-level classification, the genus name was used along with the SLC identifier as a suffix. To minimize the effects of sequencing noise, taxa with a relative abundance of < 0.1% in any given sample were filtered out from those samples in the analysis.

**Figure 2:**
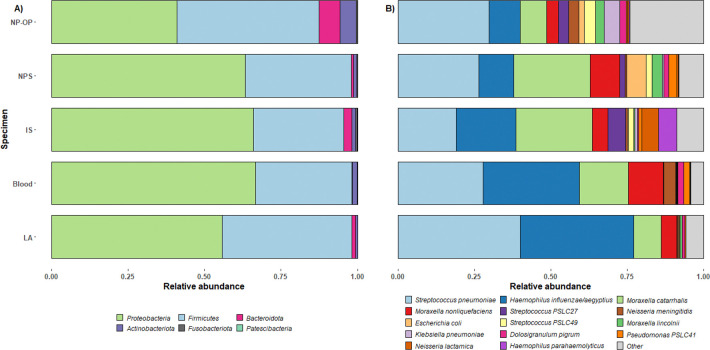
Barplots of the compositional mean relative abundances of bacteria DNA at the phylum (A) and species (B) level. All six phyla detected in our dataset are shown in the phylum-level plot (A). The Top 15 bacterial species is shown in the species-level Plot (B). The top 15 species were identified based on the sum of their compositional relative abundances across all specimen type. Specimen type is shown on the Y axis, and the compositional mean relative abundance of each taxonomic group is shown on the X axis.

The mean relative abundance of bacterial DNA at the phylum and species levels across all samples is shown in Fig. 2. The relative abundance of bacterial DNA at the same taxonomic levels for individual samples is shown in the Supplementary Information (Figure S1). Overall, the Proteobacteria (Pseudomonadota) phylum predominated in IS (66.0%), NPS (63.3%), LA (55.7%) and blood (66.6%) samples. In contrast, Firmicutes was the dominant colonizer in NP/OP samples (46.6%). Bacteroidata and Actinobacteriota contributed less than 5% of the bacterial DNA reads in all specimen types except NP/OP. Fusobacteriota was detected at low mean compositional relative abundance in NPS, NP/OP and IS (0.3% – 0.5%) samples and was absent in LA and Blood samples. Patescibacteria (Candidate Phyla Radiation) was detected in one IS sample and one NP/OP sample, with compositional relative abundances of 0.6% and 0.1% respectively in those samples. *S. pneumoniae, H. influenzae/aegyptius* and *M. catarrhalis* consistently had the highest compositional mean relative abundances across all specimen types. *M. catarrhalis* predominated in IS samples with a compositional mean relative abundance of 25.0%; *S. pneumoniae* predominated in NP/OP (29.9%), NPS (26.4%) and LA (40%) samples. In blood samples, *H. influenzae/aegyptius predominated with* 31.5%. Although *S. pneumoniae, H. influenzae/aegyptius* and *M. catarrhalis* were the most abundant based on the mean relative abundance across specimen types, they were not consistently the most dominant at the individual sample level, except in LA samples, where 57% (4/7) and 43% (3/7) of the samples had *H. influenzae/aegyptius* and *S. pneumoniae* as the dominant specie (Supplementary information, Fig. 2). We defined a species as dominant in a sample if it had the highest relative abundance in that sample. In both IS and NP/OP specimens, eight different species dominated across the 12 samples in each group.

**Figure 3:**
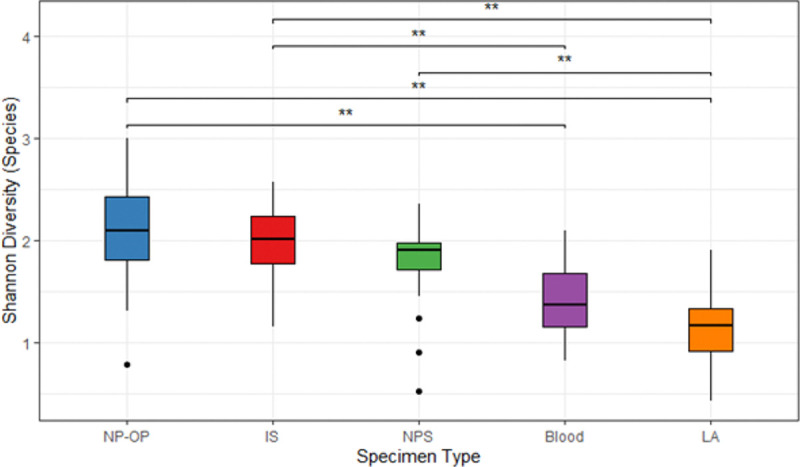
Shannon diversity compared by specimen type. Median Shannon diversity values are represented by horizontal lines within each boxplot while the upper and lower ranges of each boxplot represent the 75% and 25% quartiles. The pairwise comparison of Shannon diversity was obtained using Tukey’s Honest Significant Difference test and significant differences are indicated by an Asterix.

We compared Shannon diversity across specimen types (Fig. 3). The NP/OP samples had the highest diversity (2.13), whereas the LA samples had the lowest diversity (1.19). Pairwise comparison showed significant difference in Shannon diversity between NP/OP and LA (p = 0.0004), NP/OP and blood (p = 0.007), NPS and LA (p = 0.007), IS and Blood (p = 0.036), IS and LA (p = 0.002).

**Figure 4:**
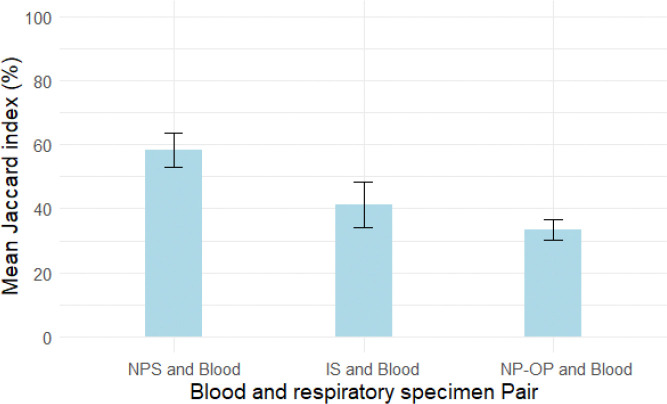
Jaccard similarity index of paired blood and respiratory body sites.

The overlap in detected species between respiratory and blood samples from the same participants was evaluated using Jaccard similarity index (Fig. 4). On average, 58% of species were shared between paired NPS and blood samples. Blood and IS shared 41% of species, while 34% of species found in the NP/OP were also found in their paired blood samples.

**Figure 5:**
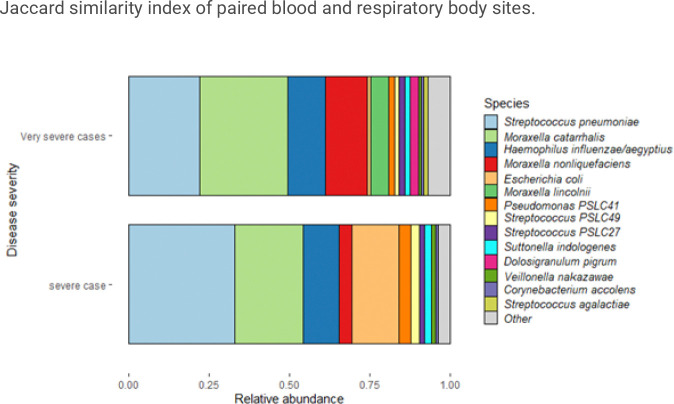
Barplots of the mean relative abundances of bacterial DNA at the species level in the nasopharynx (NPS) of severe and very severe cases. Case type is shown on the Y axis, and the relative abundance of each species is shown on the X axis.

We restricted our analyses to the nasopharynx and compared the microbial DNA profiles of children with severe and very severe pneumonia. The compositional mean relative abundance of *S. pneumoniae* in the nasopharynx was 33.1% in severe cases and 22.1% in very severe cases (Fig. 5). *M. catarrhalis* was detected at a compositional mean relative abundance of 21.3% in severe cases and 27.5% in very severe cases. The compositional mean relative abundance of *H. influenzae/aegyptius* was similar between the two groups (11.1% in severe cases vs. 11.6% in very severe cases). Notably, the relative abundance of *M. nonliquefaciens* in very severe cases (13.1%) was threefold higher than that in severe cases (3.9%). MaAsLin3 differential abundance testing of data from nasopharyngeal samples did not yield any significant association between any species and pneumonia severity classification. There was no significant difference in nasopharyngeal Shannon diversity between severe and very severe cases (p = 0.21), (Supplementary information, Figure S3). Furthermore, PERMANOVA test detected no significant difference in the nasopharyngeal microbiome composition between the two groups (P = 0.413, Supplementary information, Figure S4.)

### Metagenomic Assembled Genomes

The MAGs metrices reported follow the Minimum Information about a Metagenome-Assembled Genome (MIMAG) guidelines^26^. A total of 60 Medium-quality MAGs (≥ 50% completion and < 10% contamination) were recovered, of which 35 met the criteria for high-quality MAGs (> 90% completion and < 5% contamination) based on MIMAG standards. Details of these 35 high-quality MAGs, including their contamination and completeness as calculated using *CheckM* are provided in Table 3.

Seven species were identified from the 35 high-quality MAGs recovered. These MAGs were obtained from 22 out of 71 samples (31%), and the highest number from a single sample was three. Most MAGs were recovered from blood specimens (11/35, 31%), whereas the fewest were recovered from IS (4/35, 11%). Notably, in 83% (29/35) of the high-quality MAGs, the recovered species corresponded to the species with the highest or second highest relative abundance in the sample from which the MAG was obtained. The mean number of contigs across all 35 MAGs was 136.2 (Min:22, Max:339), and the mean contig length was 23934 bp (Min: 1500, Max: 472844; data not shown). MAG19, which had the lowest number of contigs, had the highest genome completeness at 99.7%.

Species assignment of the genomes was based on the widely used ANI threshold of ≥ 95% compared to reference genomes. MAG25 (*Limosilactobacillus sp*.) did not meet this threshold for any known bacterial species and was therefore not assigned a species-level taxonomic classification. The closest match was *Limosilactobacillus gastricus* (GCF_001434365.1), with an ANI of 83.26%. The ANI values for the remaining 42 genetically related *Limosilactobacillus* genomes in the database ranged from 76.2% to 78.9% relative to our MAG. The most common species across all MAGs were *S. pneumoniae* (11/35, 31%) and *H. influenzae* (10/35, 29%). We obtained MAGs from three unique species of *Moraxella*, including five *M. catarrhalis* MAGs.

We performed in silico serotyping and MLST on *S. pneumoniae* and *H. influenzae* MAGs. Serotype 19A was the most common *S. pneumoniae* serotype (45%, 5/11), as shown in the supplementary information (Table S2). Three of the five serotype 19A strains belonged to the same sequence type (ST847). Three *S. pneumoniae* strains could not be assigned a sequence type on the MLST database. In the only case where we recovered S. *pneumoniae* MAGs from two body sites in the same individual, the strain from blood sample was serotype 19F and ST925, while the strain from NPS was serotype 19A with an unassigned ST. All 10 of the *H. influenzae* strains were non-typeable. Five of them were assigned to distinct sequence types, while the other five could not be assigned a sequence type in the MLST database.

**Figure 6:**
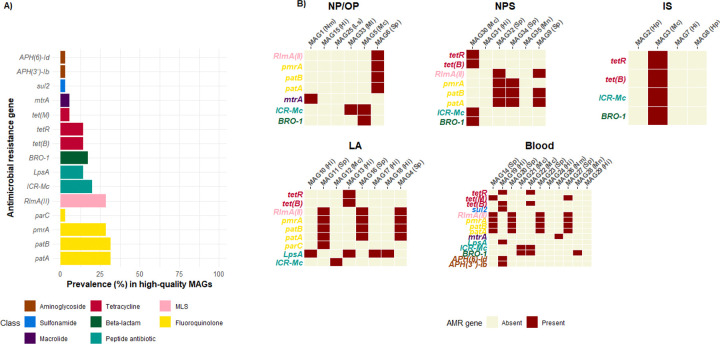
(A) Prevalence of AMR genes in all 35 high-quality MAGs. Bars are coloured according to antibiotic class of each corresponding gene. (B) AMR genes detected in the high-quality MAGs grouped by specimen type. The y axis of each plot lists all AMR genes detected within that specimen type. The AMR gene names are coloured according to their antibiotic class. MLS: Macrolide-Lincosamide-Streptogramins Nm: *Neisseria meningitidis*, Hi: *Haemophilus influenzae*, Ls: *Limosilactobacillus Sp*. Ml: *Moraxella lincolnii*, Mc: *Moraxella catarrhalis*, Sp: *Streptococcus pneumoniae*, Mn: *Moraxella nonliquefaciens*, Hp: *Haemophilus parahaemolyticus*

A total of 78 AMR genes, including 15 unique genes were detected across the 35 high-quality MAGs (Fig. 6A). These genes conferred resistance to eight antibiotic classes. AMR genes conferring resistance to fluoroquinolones were the most prevalent with four unique genes detected, followed by tetracycline resistance genes which included three unique genes across all MAGs. The average number of genes per MAG was 2.2 (range: 0–6) (Fig. 6B). Of the nine MAGs with no AMR genes detected, 78% (7/9) were *Haemophilus* species while the remaining two were MAG 25 (*Limosilactobacillus sp.*) from the NP/OP and MAG 35 (*M. lincolnii*) from the NPS.

The AMR genes were largely species specific. Up to 73% (11/15) of the unique genes were detected in a single species. The exceptions were AMR genes *tet(B), tet(R), BRO-1* and *ICR-Mc* each of which was found in MAGs from two different species. In terms of antibiotic class, AMR genes conferring resistance to tetracycline and peptide antibiotic class were present in MAGs of three different species.

Notably, all four *H. influenzae* MAGs from LA carried the *LpsA* gene while none from NPS, NP/OP and IS had the *LpsA* gene. Among the three *H. influenzae* MAGs from blood, two lacked the *LpsA* gene. All 11 *S. pneumoniae* MAGs carried the *patA* and the *patB* genes. The only non-fluoroquinolone or MLS conferring resistance genes in *S. pneumoniae* MAGs were *tet(M)* and *tet(B)* found in blood-derived MAGs. The *ICR-Mc* gene was found in all *M. catarrhalis* MAGs

## Discussion

Few studies have characterised the respiratory or blood microbiome in children with pneumonia in such detail. Using shotgun metagenomics in this pilot study, we identified *S. pneumoniae*, *H. influenzae/aegyptius* and *M. catarrhalis* were the most common species from all sample types. Furthermore, we observed species overlap of blood and respiratory samples with average Jaccard similarity index values ranging from 34% to 58%. A major strength of our work lies in the recovery of 35 high-quality MAGs in these niches, including what appears to be a novel limosilactobacillus with less than 95% ANI to any species in *GTDB-K*.

Our analysis of the microbial profile of the respiratory tract and blood during infection achieved species-level resolution which is seldom achieved in respiratory microbiome studies. Proteobacteria and Firmicutes dominated in the respiratory samples, and this was largely driven by *S. pneumoniae*, *H. influenzae* and *M. catarrhalis*. These are clinically important pathogens, and vaccines targeting *S. pneumoniae* and *H. influenzae* type b are routinely administered in many countries including The Gambia^27–29^. Our findings are consistent with other studies in The Gambia where *S. pneumoniae* and *H. influenzae* were identified as the primary bacterial pathogens causing pneumonia in children, based on microbiology culture and PCR analyses of lung and pleural aspirates^30^. The high prevalence of *S. pneumoniae* carriage observed in our analysis is also consistent with previous reports from The Gambia where carriage rates among children under one year of age were as high as 97%^31^. Although the introduction of Pneumococcal Conjugate Vaccines (PCVs) has led to the reduction of vaccine-type carriage, the overall carriage of *S. pneumoniae* remains unchanged. The reason for this persistence is likely due to the increase in the carriage of non-vaccine serotypes^32^.

*S. pneumoniae* serotype 19A was the most common serotype in our dataset among the high-quality MAGs we recovered. This serotype increased in prevalence in many countries following the introduction of the seven valent pneumococcal vaccine (PCV7)^33^. ST847 which was the most common sequence type in our data has been associated with serotype 19A in both carriage^34^ and disease^35^. *H. influenzae* type b (Hib) disease and carriage have declined globally due to the introduction of the Hib vaccine^36,37^. However, non-typeable *H. influenzae* (NTHi), which was the most common in our dataset among *H. influenzae* high-quality MAGs is now responsible for the majority of cases of otitis media, sinusitis and pneumoniae among patients that were vaccinated with the Hib vaccine^38^.

In our analysis, there were no significant differences in nasopharyngeal microbiome diversity between severe and very severe cases. Differential abundance testing also revealed no significant differences in any species by case type. This finding might be due to our small sample size given that the microbiome composition in the upper respiratory tract has been shown to serve as a marker for severity of lower respiratory tract disease^39^.

It is traditionally understood that the blood is sterile, largely from the fact that the presence of bacteria as low as 1–10 bacterial cells per millilitre of whole blood is potentially life threatening^40^. However, there is growing evidence that bacterial DNA of various taxa can be detected in blood driven by 16S or shotgun sequencing ^41–43^. It remains unclear whether bacterial DNA detected in blood represents viable organisms or remnants, and whether these arise from leakages from other body sites. Proteobacteria, Firmicutes and Actinobacteria have been reported as predominant phyla in blood across various conditions and age group^44–46^. Consistently, our analysis of children with pneumonia revealed a similar dominance of the same phyla.

The fact that DNA from many different bacterial taxa can be detected in blood poses a problem in using bacterial reads from metagenomic sequencing in determining etiology. *Barbeta, E. et al.* found 21 Operational Taxonomic Units in sixteen blood samples, even though only fifteen percent of those samples had positive blood culture^47^. A key finding from our study is the 34%–58% species overlap between respiratory and blood samples from the same individuals. Other sources of bacterial DNA in blood include the skin, or the gut with previous studies showing that a 20% overlap between bacteria Operational Taxonomic Units in blood and the gut^47^

We conducted a comprehensive sample level recovery of metagenomes resulting in the identification of 35 genomes that meet the MIMIG criteria for high-quality genomes. This was achieved using consensus binning, a very stringent approach that leverages the strengths of the different binning algorithms (*MaxBin2, MetaBAT2* and *CONCOT*) while minimising their individual limitations. This strategy recovered high-quality bins that would have been missed by using a single algorithm alone.

Recovery of MAGs from metagenomes enhances the identification of organisms that are not routinely targeted for disease diagnostic purposes by other methods such as PCR. Furthermore, it is a valuable for identifying species that are difficult to culture in the lab. Notably, we recovered a high-quality genome belonging to the limosilactobacillus genus that did not meet the 95% ANI threshold for species-level assignment in the Genome Database Taxonomy. An ANI range of 95–96% is commonly used to delineate species boundary^48,49^. Limosilactobacillus is a genus within the family lactobacillaceae. It was formally classified as lactobacillus until a reclassification in 2020 divided lactobacillus into 25 novel genera including Limosilactobacillus^50^. To date, up to 23 species of Limosilactobacillus have been described in both human and non-human hosts^51^. Our *Limosilactobacillus sp.* MAG was isolated from the NP/OP, and it potentially represents a previously undescribed or novel species within this genus.

While we used *checkM* metrices to ascertain the quality of our genomes, some studies have raised concern that the quality of MAGs may be overestimated by commonly used pipelines for assessing genome quality. *Meziti et al*.^52^ explored this by comparing gene variability between pathogenic *Escherichia coli* isolates from diarrheal samples against their corresponding MAGs recovered from the same samples. Their analysis showed that completeness estimates near 95% as reported by the *MiGA* workflow captured only 77% of the population core genes and 50% of the variable genes. They were also able to find that about 5% of the genes from the MAGs were missing in the isolate and were of different taxonomic origin, even though pipeline-based contamination estimates were as low as 1.5%. However, these findings were based on a single bacterial species and may not apply to the species present in our dataset. *Meziti et al*. further outlined key criteria that contributes to MAGs reliability beyond standard pipeline-based completion and contamination estimates. These include MAGs having fewer than 500 contigs, contig lengths greater than 1000 bp, N50 values above 20,000 bp and genome size deviations less than 0.5 Mb compared to reference strains. Our high-quality genomes meet these benchmarks in terms of contig number, contig length, genome size consistency. Additionally, more than 60% of our MAGs had N50 values exceeding 20,000 bp further supporting the overall high-quality of our MAGs. However, we still would recommend conducting gene content comparison and SNP analysis between paired *S. pneumoniae* isolate and MAG strains obtained from the same sample. This will be crucial for assessing the reliability of using sample driven *S. pneumoniae* MAGs for public health applications such as tracking vaccine impacts and in outbreak investigations. What we have not been able to achieve is the recovery of multiple high-quality MAGs of the same species from a single sample. In each case, only one strain of a single species is successfully binned. This limitation is particularly important for *S. pneumoniae* where multiple strain carriage in the upper respiratory tract is common^53,54^. Co-carriage was observed in 15% of children in South Africa^55^ using shotgun metagenomics sequencing and more than > 48% of children in Southeast Asia by microarray^56^. In our upper respiratory tract samples where *S. pneumoniae* was recovered, it is possible that more than one strain was present in some samples but only one dominant strain was recovered. This suggest that our sequencing procedure or sample-specific binning procedures are limited in their ability to recover co-existing strains of the same species in a sample. The only pair of *S. pneumoniae* MAGs recovered from blood and NPS of the same individual belonged to different serotypes and sequence types. This highlights that using MAGS to study strain-level similarity of *S. pneumoniae* across body sites may be unreliable.

While the presence of an AMR genes does not equate to clinical resistance, monitoring their presence in the respiratory tract and blood could provide insights into which antibiotics the bacteria are resistant to. Additionally, given that some AMR genes can be transferred to other bacterial species in the same niche through horizontal gene transfer, it is important to identify which genes have the potential to spread to other bacteria. The *patA* an *patB* AMR genes were highly prevalent in our *S. pneumoniae* MAGs. These genes interact to form an ATP-binding cassette (ABC) antibiotic efflux pump that confer resistance to fluoroquinolones in *S. pneumoniae*^57,58^. Resistance to the common fluoroquinolone, ciprofloxacin in *S. pneumoniae* mainly occurs through the acquisition of mutations in the quinolone resistance-determining region of the *ParC* and *gyrA* genes. However, *Lupien et al.* have shown that the *patA* and *patB* genes also contribute to low-level resistance to ciprofloxacin in a clinical isolate^59^. *M. catarrhalis* is among the species that are intrinsically resistant to colistin. The ICR-Mc gene found in our *M. catarrhalis* MAGs encodes a chromosomally located colistin resistance phosphoethanolamine (PEtN) transferase^60^. The protein is the closest known ortholog to the well-known plasmid mediated colistin resistance genes *mcr-1* and *mcr-2*^60^. The *LpsA* gene was detected in *H. influenzae* MAGs in LA but absent in MAGs from NP/OP, NPS and IS. *LpsA* confers intrinsic resistance to peptide antibiotics^61^ but it is more widely known for its role in lipooligosaccharide biosynthesis^62^ and it has been associated with adaptation to the lung environment and invasiveness^63^

A limitation of our study is the sample size and therefore some of our findings are limited by insufficient statistical power and generalisability. Secondly, we did not obtain controls at the sampling point to control for contamination during sampling. However, we were able to minimise this effect by excluding taxa corresponding to *Cutibacterium acnes, Staphylococcus saprophyticus* and *Staphylococcus epidermidis* from all samples in this analysis. This decision was made because these bacteria primarily colonise the skin and not the specimen types we analysed and could have contaminated our samples during sample collection. They have also been implicated as contaminants in microbiome studies of low-biomass samples^40,64^. A third limitation is that some children were administered antibiotics before sample collection, and this could have impacted their microbiome profiles. Finally, our analysis couldn’t establish whether the relative abundances of the different species in our samples reflected the existence of viable bacteria or bacteria-derived DNA. Therefore, the bacterial profiles of samples may not represent the viable microbiome at the time of sample collection, and as such our findings should be interpreted with caution.

## Conclusion

Despite our limitations, we have been able to show that overall, bacterial DNA from *S. pneumoniae, H. influenzae/aegyptius* and *M. catarrhalis* were the most common across the different body sites we studied in children with pneumonia in The Gambia. We have also shown an overlap in DNA from bacterial taxa present in the respiratory tract and blood. In addition, we have been able to recover high quality MAGs from our samples further advancing the field of genomics of important pathogens like *H. influenzae and S. pneumoniae.* For future studies, it is essential to include sampling controls that undergo the same sequencing procedure as the samples. We also propose future studies in metatranscriptomics to study the activities of live bacteria present in blood and respiratory samples of pneumonia patients. Finally, we propose comparing MAGs and isolates of *S. pneumoniae* strains from the same respiratory or blood sample to assess how well the MAG genome reflects that of the isolate.

## Methods

### Study participants and sample collection

We used stored samples obtained between 2011 and 2014 from participants recruited in the Pneumonia Etiology Research for Child Health (PERCH) study^65^. PERCH was a case-control study that sought to characterise the causes of severe childhood pneumonia in children living in high pneumonia burden and low-resource regions, including The Gambia. The samples analysed in this study were exclusively from children with pneumonia. In the PERCH study, children aged 1–59 months who presented and were admitted to the hospital in Basse, Upper River Region of The Gambia, with severe or very severe pneumonia were recruited. Severe pneumonia was defined as cough or difficulty breathing with lower chest wall indrawing, and very severe pneumonia was defined as cough or difficulty breathing and at least one of the following signs: vomiting everything, difficulty in drinking or breastfeeding, central cyanosis, lethargy, convulsions, unconsciousness, or head-nodding. Specimens, including NPS, NP/OP swabs, blood, IS and LA, were collected at enrolment. Further details on participant recruitment, including case definitions, inclusion criteria and sample collection procedures of the PERCH study are described elsewhere^65,66^.

### Ethics

Ethical approval for the Gambian samples used in the PERCH study was obtained from the Johns Hopkins Bloomberg School of Public Health Institutional Review Board (JHSPH IRB), and local ethical approval was obtained from the Joint MRC Unit The Gambia at The London School of Hygiene and Tropical Medicine and The Government of The Gambia Ethics Committee. Written informed consent was obtained from the patient’s guardians for all procedures performed in the study. All procedures were performed in accordance with relevant guidelines and regulations.

### Laboratory procedures

DNA was extracted from all samples using QIAGEN kits, following the manufacturer’s instructions. Total DNA was extracted from the stored NPS, NP/OP, and IS samples using the DNeasy PowerSoil Kit^67^, whereas DNA from the LA and blood samples was extracted using the QIAamp MinElute Virus Spin kit^68^ and the QiAamp DNA Blood Mini Kit^69^, respectively. The laboratory subscribed to a Canadian External Quality Assessment (EQA) provider. Extracted DNA samples were enriched for microbial DNA using the NEBNext Microbiome DNA kit (New England Biolabs) following the manufacturer’s instructions before whole metagenomic sequencing using the Illumina NovaSeq 2X150bp. Blank controls (sterile swabs) were included throughout the library preparation process to monitor for contamination in the laboratory reagents.

### Sequencing reads processing

Sequencing reads were mapped to the human reference sequence (T2T), and all matching reads were removed. Quality assessment of reads and the recovery and classification of metagenomes were performed using modules in the Viral (micro) Eukaryotic Bacterial Archaeal (VEBA) open-source software suite^70,71^. Adapter removal and quality trimming were performed using the *preprocess.py* module, and paired trimmed reads were assessed and corrected using the *repair.sh* module of *BBTools.* Cleaned reads were input into the *assembly.py* module, where they were assembled using *SPAdes*-based assemblers. Binning was done using *MetaBAT2*^72^, *CONCOCT*^73^, and *MaxBin2*^74^ followed by *DAS* Tool^75^ for consensus binning. Metagenome-assembled genomes (MAGs) were quality assessed by *CheckM*^76^. The genome statistics such as N50, number of contigs and genome size were calculated using *SeqKit*^77^. Taxonomic classification of the genomes was performed using the Genome Taxonomy Database Toolkit *GTDB-TK*^78^ based on sequence similarity (FASTANI) and phylogenetic placement.

MAGs with ≥95% Average Nucleotide Identity (ANI) were clustered to form a species-level cluster (SLC). Sequencing reads were mapped to SLCs representative genomes, and counts were generated using *featureCounts* as part of the *coverage.py* module. For samples with unbinned contigs, pseudo- coassemblies were created to enable additional binning and taxonomic assignment.

### *S. pneumoniae* and *H. influenzae* serotyping and Multi-Locus Sequence Typing

FASTA files from *S. pneumoniae* MAGs were analysed using PneumoKITy^79^ to assign in silico *S. pneumoniae* serotypes. In silico serotyping of *H. influenzae* MAGs was performed using hicap^80^. Multi-locus sequence types for both *S. pneumoniae* and *H. influenzae* were assigned using PubMLST^81^. The seven housekeeping genes analysed for *S. pneumoniae* were: *aroE, ddl, gdh, gki, recP, spi,* and *xpt.* For *H. influenzae,* the seven genes were: *adk, atpG, frdB, fucK, mdh*, *pgi* and *recA*

### Antimicrobial Resistance determinants for MAGs

The recovered MAGs were submitted to the Comprehensive Antimicrobial Resistance Database (CARD) to identify and annotate AMR genes. Only AMR gene hits classified under “perfect” or “strict” categories and a cutoff of >90% sequence identity of the matching region were considered.

### Statistical analysis

Statistical analysis and visualisation were conducted using R studio (version 2024.12.0.467). We applied a one-way analysis of variance (ANOVA) to compare Shannon diversity indices between specimen types and applied Tukey’s Honest Significant Difference test for pairwise comparison of specimen types. The Jaccard similarity index was calculated to assess bacterial species overlap in paired blood and respiratory samples.

Kruskal-Wallis test was used to compare Shannon diversity between the nasopharynx of severe cases and very severe cases. We computed the Aitchison distance (i.e., Euclidean distance on the centered log-ratio transformed data) and used principal coordinates analysis (PCoA) to plot the beta diversity of the nasopharynx in severe and very severe cases. Permutational multivariate analysis of variance (PERMANOVA) was performed to determine whether beta diversity differed between the two groups.

Differential abundance testing was performed to identify differences in microbial species between the nasopharynx of patients with severe and very severe disease using Microbiome Multivariable Associations with Linear Models (MaAsLin3)^82^, accounting for both taxa prevalence and abundance. We controlled for age and applied Benjamini-Hochberg False Discovery Rate (FDR) for multiple testing correction (q value). The q value threshold for significance was set to the default <0.1 for MaAsLin3. All figures were generated using ggplot2 and microViz^83^.

## Supplementary Material

Supplementary Files

This is a list of supplementary files associated with this preprint. Click to download.

• Supplemetaryinformationupdated.pdf

## Figures and Tables

**Table 1:** Demographic and clinical characteristics of children. This table includes all children who contributed at least one specimen to any analysis.

	Classification	Frequency (%)
Sex	Male	22 (59)
	Female	15 (41)
Age	1–5 months	15 (41)
	6–11 months	4 (11)
	12–23 months	8 (22)
	24–59 months	10 (27)
Hib vaccination status	Not vaccinated	6 (16)
	Partially vaccinated	9 (24)
	Fully vaccinated	20 (54)
	No data	2 (5)
PCV vaccination status	Not vaccinated	8 (22)
	Partially vaccinated	8 (22)
	Fully vaccinated	19 (51)
	No data	2 (5)
Pneumonia severity	Severe	16 (43)
	Very severe	21 (57)
HIV status	Positive	2 (5)
	Negative	31 (84)
	Unconfirmed	4 (11)

**Table 2:** Characterisation of high-quality metagenome-assembled genomes obtained from blood and respiratory samples

	Specie	ANI with type strain (%)	Number of contigs	N50	Genome Size (Mb)	Completeness (%)	Contamination (%)	Strain heterogeneity	Source specimen
MAG1	*Neisseria meningitidis*	97.58	227	11345	1.80	95.79	0.23	0	NP-OP
MAG2	*Haemophilus parahaemolyticus*	97.3	146	17420	1.78	90.94	1.24	66.67	IS
MAG3	*Moraxella catarrhalis*	99.12	273	8974	1.75	92.31	1.38	22.22	IS
MAG4	*Streptococcus pneumoniae*	98.53	114	26698	2.00	98.5	0	0	LA
MAG5	*Moraxella catarrhalis*	99.08	69	38190	1.77	95.76	0.42	33.33	NP-OP
MAG6	*Streptococcus pneumoniae*	98.62	69	44339	2.16	99.63	0.06	0	NP-OP
MAG7	*Haemophilus influenzae*	97.17	96	50042	1.92	99.37	0.25	50	IS
MAG8	*Haemophilus parahaemolyticus*	97.24	265	9977	1.83	90.75	1.05	28.57	IS
MAG9	*Streptococcus pneumoniae*	98.63	244	11618	1.80	93.6	0.41	20	NPS
MAG10	*Haemophilus influenzae*	97.14	56	93732	1.84	99.55	0.76	16.67	LA
MAG11	*Streptococcus pneumoniae*	99.52	76	67168	2.02	99.63	0	0	LA
MAG12	*Moraxella catarrhalis*	96.14	175	28060	2.00	97.56	3.54	10.53	LA
MAG13	*Haemophilus influenzae*	97.49	247	11400	1.83	92.84	3.45	56.52	LA
MAG14	*Streptococcus pneumoniae*	98.6	88	32376	2.05	99.63	0	0	Blood
MAG15	*Haemophilus influenzae*	97.39	167	20433	1.63	91.37	0.8	100	NP-OP
MAG16	*Streptococcus pneumoniae*	98.57	87	39492	2.07	99.44	0	0	LA
MAG17	*Haemophilus influenzae*	97.1	60	71164	1.80	98.98	0	0	LA
MAG18	*Haemophilus influenzae*	97.38	29	122554	1.79	99.55	0	0	LA
MAG19	*Haemophilus influenzae*	98.8	22	137938	1.85	99.66	0.23	0	Blood
MAG20	*Streptococcus pneumoniae*	98.45	197	16266	2.01	97.44	1.07	0	Blood
MAG21	*Moraxella catarrhalis*	99.23	139	28747	1.94	97.29	0.63	75	Blood
MAG22	*Moraxella catarrhalis*	99.12	26	120380	1.85	99.45	1.07	14.29	Blood
MAG23	*Streptococcus pneumoniae*	98.65	62	45294	2.08	99.63	0	0	Blood
MAG24	*Haemophilus influenzae*	97.11	26	186272	1.86	99.55	0	0	Blood
MAG25	*Limosilactobacillus Sp.*		228	8961	1.50	97.81	0.13	0	NP-OP
MAG26	*Neisseria meningitidis*	97.61	158	18553	1.97	99.18	0.38	0	Blood
MAG27	*Streptococcus pneumoniae*	98.56	64	48015	2.12	99.63	0	0	Blood
MAG28	*Moraxella nonliquefaciens*	98.47	56	343360	2.28	96.85	3.48	0	Blood
MAG29	*Haemophilus influenzae*	97.21	157	29106	1.93	95.55	1.05	28.57	Blood
MAG30	*Moraxella catarrhalis*	99.08	65	74719	1.98	98.88	0.44	25	NPS
MAG31	*Haemophilus influenzae*	97.28	156	19152	1.75	98.05	0.84	40	NPS
MAG32	*Streptococcus pneumoniae*	98.59	339	6926	1.83	91.90	1.37	83.33	NPS
MAG33	*Moraxella lincolnii*	97.95	243	13659	2.07	92.60	0.5	66.67	NP-OP
MAG34	*Streptococcus pneumoniae*	98.86	238	10781	1.90	94.76	0.75	50	NPS
MAG35	*Moraxella nonliquefaciens*	98.22	103	57578	2.34	95.75	4.84	6.06	NPS

## Data Availability

Shotgun metagenomic sequencing data has been deposited in the NCBI Sequence Read Archive associated with BioProject PRJNA727021. The accession numbers of the 71 samples used in the analysis in this paper are detailed in Supplementary Table S3. The genome assemblies of the 35 high-quality MAGs reported in this paper have been deposited in the Zenodo repository under https://zenodo.org/records/18494646. Other data will be made available on request
